# Significance of the immunofluorescence staining patterns and titres of the antinuclear antibody test in paediatric rheumatology setting

**DOI:** 10.55730/1300-0144.5572

**Published:** 2023-01-17

**Authors:** Özge BABA, Hakan KISAOĞLU, Mukaddes KALYONCU

**Affiliations:** Division of Pediatric Rheumatology, Department of Pediatrics, Faculty of Medicine, Karadeniz Technical University, Trabzon, Turkey

**Keywords:** Antinuclear antibody, ANA associated diseases, dense fine speckled pattern, immunofluorescence staining pattern, titre

## Abstract

**Background/aim:**

Antinuclear antibody (ANA) is among the most frequently ordered tests in paediatric rheumatology setting. Diseases like systemic lupus erythematosus and Sjögren syndrome is closely related with a positive ANA and classified as ANA associated diseases. Besides, ANA test is ordered in patients with juvenile idiopathic arthritis (JIA) to assess the risk for uveitis and a positive ANA could be detected in children with nonrheumaticrheumatic conditions. In this study, we aimed to investigate frequency of positive ANA in paediatric rheumatology setting and the association of immunofluorescence staining patterns and titres of ANA with rheumatic diseases.

**Materials and methods:**

Immunofluorescence staining patterns, and titres of the ANA and diagnoses of children who tested for ANA between January 2016 and December 2021 were retrospectively analysed.

**Results:**

Among 2477 patients with ANA tested, 28.1% had a positive ANA result. Among them, 39.2% had a diagnosis of a rheumatic disease. Most common rheumatic diagnosis was JIA (43.8%) and ANA associated diseases were observed in 24.5% of the patients with a rheumatic diagnosis. While ANA associated diseases had significantly more frequent homogenous staining, dense fine speckled pattern was significantly more common in children with nonrheumatic diagnoses. Despite ANA associated diseases was found to be significantly associated with higher titres, no difference was observed between patients with JIA and nonrheumatic conditions.

**Conclusion:**

Our study showed that the majority of children with a positive ANA test were not diagnosed with a rheumatic disease. While titres and patterns of ANA were found to be important in diagnosis of rheumatic diseases, ordering ANA test with solid indications might give improved probability of rheumatic diagnoses in children with a positive test.

## 1. Introduction

The term of antinuclear antibody (ANA) refers to any of a large group of autoantibodies that recognise predominantly, but not always specifically, cellular antigens in the cell nucleus. They are antibodies that develop against structures such as DNA, histones, and centromeres [1]. Presence of antinuclear antibodies (ANAs) is associated with various systemic rheumatic diseases, including systemic lupus erythematosus (SLE), systemic sclerosis, primary Sjögren syndrome, mixed connective tissue disease and idiopathic inflammatory myopathies (such as polymyositis and dermatomyositis). These diseases are collectively referred to as ANA associated rheumatic diseases, and several autoantibodies that are specific to each disease have been identified [2]. However, without any signs of disease, ANA could be detected in healthy people and observed 4%–15% of healthy children [3–5]. Besides, a positive ANA test might be observed in malignant and infectious diseases [6,7] and has a high rate of false-positive results for rheumatic diagnoses [8].

The American College of Rheumatology ANA Task Force position statement recommended the indirect immunofluorescence assay (IFA) using HEp-2 substrate as the “gold standard” for primary ANA detection [9]. However, some clinical laboratories use solid-phase immunoassays, in some cases as a reflex test to supplement HEp-2 IFA screening test, or even replace HEp-2 IFA testing. Nevertheless, most clinical laboratories worldwide depend heavily on HEp-2 IFA as the primary screening method. The ANA staining pattern raises suspicion for various diseases and helps clinicians to perform confirmatory tests with clinical basis [9].

Positive ANA and high titres have been reported to be associated with a diagnosis of SLE in children but, no diagnostic utility has been shown in children with JIA [10]. Besides, positive ANA alone was suggested as a poor indicator for a rheumatic diagnosis in children [11].

In this study, we aimed to investigate the frequency of the positive ANA test in paediatric rheumatology setting and the association of the immunofluorescence staining patterns and titres of ANA with rheumatic diseases.

## 2. Materials and methods

Medical charts of children, evaluated in the paediatric rheumatology clinic between January 2016 and December 2021, in whom at least one ANA test was ordered, were reviewed. Patients with a positive ANA at least in one occasion were included. Positive ANA was defined as ≥1/80 titre in indirect IFA on Hep-2 cell substrates. Patients with a positive ANA, with immunofluorescence staining patterns of ANA not reported according to The International Consensus on ANA-staining Patterns (ICAP) recommendations [12], were excluded. Age, sex, and diagnosis of the patients were collected from the medical charts. Among patients, association of the titres and patterns of ANA were investigated in patients with JIA, ANA associated diseases and nonrheumatic conditions.

Immunofluorescence staining patterns of ANA were investigated under 4 major groups, according to the ICAP recommendations [12]. While homogenous and dense fine speckled (DFS) patterns were assessed as sole patterns, fine and coarse speckled patterns were classified under speckled. Other nuclear staining patterns, such as centromere, nucleolar and discrete nuclear dots, were classified under the other nuclear group. Cytoplasmic and mitotic staining patterns were not investigated due to the rare detection rate. Among patients with a positive ANA more than one occasion, staining pattern of the highest titre were taken into account. ANA titres were classified as 1+ in titres between 1/80 and 1/320, 2+ in titres between 1/320 and 1/1000, 3+ in titres between 1/1000 and 1/3200 and 4+ in titres > 1/3200 due to the laboratory preference of the ANA results.

In statistical evaluation, data obtained by measurement are shown as mean ± standard deviation, and data obtained by counting are shown as percentage. The normal distribution of data was analysed by using the Kolmogorov-Smirnov test. One-way Anova or Kruskal-Wallis test were used for the analyses of quantitative data between three groups according to the distribution of the data. The Kruskal-Wallis test followed by Dunn’s posthoc test was used to compare the groups according to age at positive ANA. Chi-square test was used for the comparison of qualitative data. Posthoc analysis for the significant chi-square values was carried out by calculating the significant adjusted residuals. The level of significance was set at p value < 0.05.

## 3. Results

Between January 2016 and December 2021, ANA test was ordered in 2477 patients and a positive ANA was reported in 697 (28.1%) of them. Among ANA positive patients, only 273 (39.2%) were diagnosed with a rheumatic disease. Most common diagnosed rheumatic disease was JIA in 120 (43.8%) of the patients followed by SLE in 50 (18.2%) and vasculitis in 46 (16.8%). Among patients with JIA, most common subtype was persistent oligoarticular JIA in 91 (75.8%) followed by polyarticular JIA in 16 (13.3%), extended oligoarticular in 14 (11.7) patients. Enthesitis related arthritis, psoriatic arthritis and systemic onset JIA were the least frequent diagnoses in ANA positive patients with JIA. ANA associated diseases including SLE, juvenile dermatomyositis, Sjögren syndrome and scleroderma was observed in 67 (24.5%) of the patients. Among patients with vasculitis most frequent diagnosis was immunoglobulin A vasculitis in 36 (78.3%) of the patients. Behçet’s disease, Takayasu arteritis, polyarteritis nodosa and hypocomplementemic urticarial vasculitis were the other diagnoses in patients with positive ANA. Since positive ANA is not implicated in pathogenesis and clinical findings in patients with vasculitis and heterogeneity of the pathogenesis among different vasculitis types, we did not include patients with vasculitis in comparative analyses. Flow chart of the study population and distribution of the rheumatic diseases are shown in Figure and Table 1, respectively.

Comparison of the age and sex according to the diagnosis of the patients revealed a higher age at ANA testing (mean age in ANA associated diseases: 12.5 ± 3.5, JIA: 9.4 ± 4.6 and nonrheumatic conditions: 9.7 ± 3.9, p: <0.0001) and a higher frequency of female sex (ANA associated diseases 85.1%, JIA 68.4% and nonrheumatic conditions 62.2%, p: 0.001) in ANA associated diseases than patients with JIA and nonrheumatic conditions.

In patients with ANA associated diseases, the most reported ANA pattern was homogenous in 34.3% of the patients. While spotted ANA patterns were the most observed pattern in patients with JIA (28.3%), DFS staining pattern was the most frequently reported pattern in patients with nonrheumatic conditions (34.8%). Comparison of the staining patterns among patients revealed a significant trend towards increased frequency of DFS pattern (ANA associated diseases 10.4%, JIA 21.7% and nonrheumatic conditions 34.8%, p < 0.0001) and decreased frequency of homogenous staining (ANA associated diseases 34.3%, JIA 26.7% and nonrheumatic conditions 8.9%, p < 0.0001) in nonrheumatic conditions. Assessment of the ANA titres revealed a significantly more frequent high ANA titres (>1/1000) in ANA associated diseases compared to the patients with JIA and nonrheumatic conditions (ANA associated diseases 53.7% vs. JIA 16.7% and nonrheumatic conditions 11.6%, p < 0.0001). However, distribution of the titres between patients with JIA and nonrheumatic conditions did not significantly differ among patients (p > 0.05). Comparison of patient characteristics, ANA staining pattern and titres among patients with ANA associated diseases, JIA and nonrheumatic diseases is given in Table 2.

## 4. Discussion

In our study, the majority of the children who tested positive for ANA did not have a rheumatic diagnosis and JIA was the most common rheumatic disease in children with a positive ANA result. Similar to our results, JIA was the most common rheumatic diagnosis in children with a positive ANA in another study [13]. In contrast, an earlier study found nonrheumatic conditions in 27% of the children with a positive ANA and showed that majority of the children who have positive ANA test without any autoimmune diagnosis at initial diagnosis will not develop an autoimmune condition [14]. This might be associated with increased referral of patients to the rheumatology departments and increased usage of ANA testing without solid indications. A similar observation was reported by Haslak et al. [11], and in their study majority of the patients (94.1%) referred to paediatric rheumatology clinic for a positive ANA had no underlying disease and none of them developed any autoimmune conditions or ANA associated rheumatic diseases.

In our study, homogenous staining pattern and higher titres were more frequently detected in ANA associated diseases and majority of the patients with ANA associated diseases diagnosed with SLE. SLE is a prototypic autoimmune disease, and immunological hallmark is the production of ANA [15]. Higher titres, presence of multiple autoantibodies and homogenous staining pattern were shown to be associated with a diagnosis of SLE [16,17].

ANA testing is often considered in paediatric patients presenting with joint pain to determine the possibility of an alternative diagnosis to JIA or systemic autoimmune related diseases such as SLE. A positive-ANA has been reported in 30%–50% of JIA patients in varying proportions across the JIA subtypes [18]. An elevated ANA titre has been reported in all JIA subtypes although is most prevalent in oligoarticular JIA (persistent and extended) [19]. The detection of ANA is important because presence determines the frequency of ocular assessment for asymptomatic uveitis [20]. Traditionally, ANA has not been used as an aid in the diagnosis of JIA, but as a risk biomarker for developing uveitis [19]. Uveitis is the most common of extraarticular manifestations in JIA and can have a significant impact on morbidity if detection and treatment of uveitis is delayed [21]. The most commonly reported and most sensitive cut-off titre for JIA was 1/80, although there was a large variation in published ANA immunofluorescence serum dilutions (1/40–1/320) used for laboratory investigation [18]. Although previous studies have shown that ANA titres were not significantly different from in patients with JIA than nonrheumatic conditions [22], no study investigated the staining patterns between JIA and healthy controls. In our study, except for higher homogenous pattern in patients with JIA, none of the staining patterns significantly differed between children with nonrheumatic conditions and JIA. In a Nordic study, antihistone antibodies were found to be significantly associated with JIA uveitis [23] and antihistone antibodies were expected to be stained as homogenous which might partially explain the observation of higher homogenous staining pattern in patients with JIA in our study. Also, a recent study reported a more frequent homogenous pattern of ANA in children with uveitis [24].

The staining pattern of ANA may provide clues about diseases. For example, the homogeneous nuclear pattern frequently associated with SLE while fine granular mottled pattern observed more common in Sjögren’s disease [25]. It has been reported that some patterns of ANA staining are associated with certain nuclear antigens that are related to particular manifestations of specific diseases [12]. As there are many possible nuclear antigens, ANA are classified into specific autoantibodies using different techniques such as immunoblotting or enzyme-linked immunosorbent assay such as anti-dsDNA, anti-Sm, anti-SSA/Ro and myositis specific antibodies.

In our study, DFS staining pattern was more frequently reported in children with nonrheumatic conditions. In the absence of any disease specific antibodies, a positive DFS pattern with a positive anti-DFS70 antibody is unlikely to be associated with systemic autoimmune disease [26,27]. In a recent study, only half of the children with a positive ANA with DFS pattern exhibited positive anti-DFS70 antibodies. In addition, anti-DFS70 antibodies were less likely to be found positive in children with autoimmune diseases and in all children with a positive anti-DFS70 antibody with an autoimmune disease, a disease specific antibody was observed [24]. Thus, evaluation of disease specific and anti-DFS70 antibodies along with ANA test might be more accurate than the assessment of the immunofluorescence pattern of ANA alone in children with suspected autoimmune disease.

Retrospective design is the main limitation of our study. Besides, this study did not include the follow-up data of patients with nonrheumatic conditions which might overestimate the prevalence of nonrheumatic conditions in ANA positive children. Despite immunofluorescence patterns of ANA was reported in a single laboratory, interpretation of the staining patterns might not be standardised. Another point to consider is the pretest probability of rheumatic disease in ANA tested children. Also presence of uveitis and association with pattern and titre of ANA was not investigated which might be regarded as a limitation. A higher pretest probability results with a higher predictive value [28]. And that might result in different predictive value of ANA test in a different rheumatology setting.

In conclusion, the majority of the children with a positive ANA did not have a rheumatic disorder. Ordering ANA test with more solid indications might result in an increased sensitivity for rheumatic diseases. Despite homogenous staining pattern and higher titres of ANA were associated with ANA associated diseases, presence of autoimmune diseases in patients with DFS pattern ANA suggests that interpretation of ANA test might be more accurate in the presence of specific antibody panels.

## Figures and Tables

**Figure:**
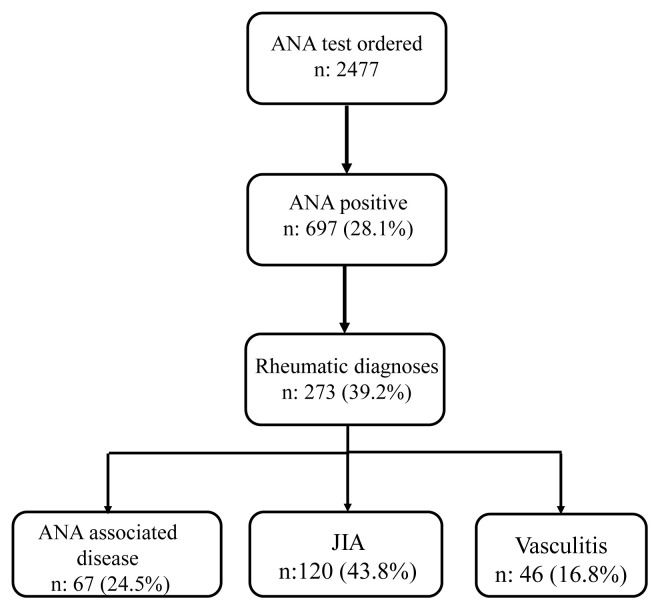
Flowchart of the study population **(**ANA: antinuclear antibody, JIA: juvenile idiopathic arthritis, n: number).

**Table 1:** Distribution of the diagnoses of children with rheumatic disease in children with a positive ANA result.

Diagnosis	n (%)
JIA	120 (43.8)
SLE	50 (18.2)
Vasculitis	46 (16.8)
Sjögren syndrome	7 (2.6)
Scleroderma	7 (2.6)
Dermatomyositis	3 (1.1)

ANA: antinuclear antibody, n: number, JIA: juvenile idiopathic arthritis, SLE: systemic lupus erythematosus.

**Table 2:** The comparison of the IF patterns and titres of antinuclear antibody test according to the diagnoses.

	ANA associated diseases	JIA	Nonrheumatic conditions
	n: 67 (%)	n: 120 (%)	n: 423 (%)

**Age**, mean±SD	12.5 ± 3.5**	9.4 ± 4.6	9.7 ± 3.9

**Sex (female)**	57 (85.1%)	82 (68.4%)	263 (62.2%)
**Adj. res.**	3.3*	0.7	−2.8*

**ANA pattern**			

**Dense fine speckled**	7 (10.4%)	26 (21.7%)	147 (34.8%)
**Adj. res.**	−3.7**	−2.0**	4.2**

**Homogeneous**	23 (34.3%)	32 (26.7%)	38 (8.9%)
**Adj. res.**	4.5**	4.0**	−6.5**

**Speckled**	21 (31.3%)	34 (28.3%)	131 (30.9%)
**Adj. res.**	0.1	−0.5	0.3

**Other nuclear**	13 (19.4%)	22 (18.4%)	101 (23.9%)
**Adj. res.**	−0.7	−1.1	1.4

**ANA titres**			

**(+)**	15 (22.4%)	66 (55.0%)	253 (59.8%)
**Adj. res.**	−5.6**	0.1	3.8**

**(++)**	16 (23.9%)	34 (28.3%)	115 (27.2%)
**Adj. res.**	−0.6	0.4	0.1

**(+++)**	13 (19.4%)	13 (10.8%)	45 (10.6%)
**Adj. res.**	2.1**	−0.3	−1.2

**(++++)**	23 (34.3%)	7 (5.8%)	10 (2.4%)
**Adj. res.**	9.7**	−0.4	−6.3**

IF: immunofluorescence, ANA: antinuclear antibody, JIA: juvenile idiopathic arthritis, n: number, SD: standard deviation.

* p <0.01

** p < 0.001.
